# Plant-Produced Chimeric V_H_H-sIgA Against Enterohemorrhagic *E. coli* Intimin Shows Cross-Serotype Inhibition of Bacterial Adhesion to Epithelial Cells

**DOI:** 10.3389/fpls.2019.00270

**Published:** 2019-03-12

**Authors:** Reza Saberianfar, Adam Chin-Fatt, Andrew Scott, Kevin A. Henry, Edward Topp, Rima Menassa

**Affiliations:** ^1^Agriculture and Agri-Food Canada, London Research and Development Centre, London, ON, Canada; ^2^Department of Biology, University of Western Ontario, London, ON, Canada; ^3^Human Health Therapeutics Research Centre, National Research Council Canada, Ottawa, ON, Canada

**Keywords:** EHEC, SIgA, V_H_H, chimeric antibody, molecular farming

## Abstract

Enterohemorrhagic *Escherichia coli* (EHEC) has consistently been one of the foremost foodborne pathogen threats worldwide based on the past 30 years of surveillance. EHEC primarily colonizes the bovine gastrointestinal (GI) tract from which it can be transmitted to nearby farm environments and remain viable for months. There is an urgent need for effective and easily implemented pre-harvest interventions to curtail EHEC contamination of the food and water supply. In an effort to address this problem, we isolated single-domain antibodies (V_H_Hs) specific for intimin, an EHEC adhesin required for colonization, and designed chimeric V_H_H fusions with secretory IgA functionality intended for passive immunotherapy at the mucosal GI surface. The antibodies were produced in leaves of *Nicotiana benthamiana* with production levels ranging between 1 and 3% of total soluble protein. *in vivo* assembly of all subunits into a hetero-multimeric complex was verified by co-immunoprecipitation. Analysis of multivalent protection across the most prevalent EHEC strains identified one candidate antibody, V_H_H10-IgA, that binds O145:Hnm, O111:Hnm, O26:H11, and O157:H7. Fluorometric and microscopic analysis also indicated that V_H_H10-IgA completely neutralizes the capacity of the latter three strains to adhere to epithelial cells *in vitro*. This study provides proof of concept that a plant-produced chimeric secretory IgA can confer cross-serotype inhibition of bacterial adhesion to epithelial cells.

## Introduction

Consumption of enterohemorrhagic *Escherichia coli* (EHEC) via contaminated food or water is associated with intestinal hemorrhage and osmotic dysregulation (Kandel et al., 1989). Each year, EHEC is estimated to affect approximately 230,000 people in the United States and is the fourth most frequently isolated food-borne pathogen from clinical stool samples (Hale et al., 2012). Approximately 73,000 EHEC infections are caused by the O157:H7 serotype which has consistently been the most prevalent and virulent EHEC serotype over the approximately 30 years of United States national surveillance (Hale et al., 2012). Six additional EHEC serogroups, O26, O45, O103, O111, O121, and O145, known as the “Big Six,” generally comprise >90% of non-O157 infections during any given year and have been traced to at least 22 human disease outbreaks in the United States since 1990 (CDC, 2017).

The gastrointestinal (GI) tract of cattle is considered the primary reservoir of EHEC and can contaminate various food or water supplies via excreted fecal matter or after slaughter during processing of the carcass (Montenegro et al., 1990; Beutin et al., 1993). Indeed, cattle density has been identified as a primary risk factor for the incidence of local EHEC infections (Brehony et al., 2018). In accord with the “One Health” framework, virtually all strategic interventions to prevent EHEC transmission to humans have focused on minimizing colonization of cattle reducing the risk of contamination from fecal shedding or at harvest. In cattle, EHEC principally adheres to and colonizes the lymphoid follicle-dense mucosa at the terminal rectum known as the rectoanal junction (Phillips et al., 2000; Naylor et al., 2003; Lim et al., 2007). The adhesin protein known as intimin mediates interaction of the bacteria with uninfected host epithelial cells and is a necessary prerequisite for intimate bacterial adhesion and colonization (Frankel et al., 1994).

The use and efficacy of recombinant secretory immunoglobulin A (sIgA) in passive mucosal immunotherapy is well established (Enriquez and Riggs, 1998; Virdi et al., 2013; Nakanishi et al., 2017; Vanmarsenille et al., 2018). Because sIgA application can impart immediate, albeit transient, protection from a pathogen, it may be of value to beef producers and processors as a pre-harvest intervention for EHEC. In the GI tract, sIgA primarily functions to clear pathogens by immune exclusion: after binding to its target, glycans on the secretory component (SC) facilitate binding to the mucus lining of the GI tract enabling clearance of sIgA–pathogen complexes by peristalsis (Macpherson et al., 2008). A sIgA directed against intimin would thus be expected to prevent luminal EHEC cells from interacting with the host epithelium, clearing them by entrapment in the mucus layer and subsequent fecal shedding.

Structurally, sIgA consists of an IgA dimer linked by two additional chains: a 15-kDa joining chain (JC) that links the IgA Fc end-to-end (Krugmann et al., 1997) and a 70-kDa SC that coils around both Fc chains (Bonner et al., 2007). A plant production platform is currently the most suitable for producing recombinant sIgA because of the requirement for glycosylation and disulfide bond formation for proper folding and assembly of sIgA subunits as well as higher relative yields and the prospect of oral delivery (Wycoff, 2005).

**FIGURE 1:**
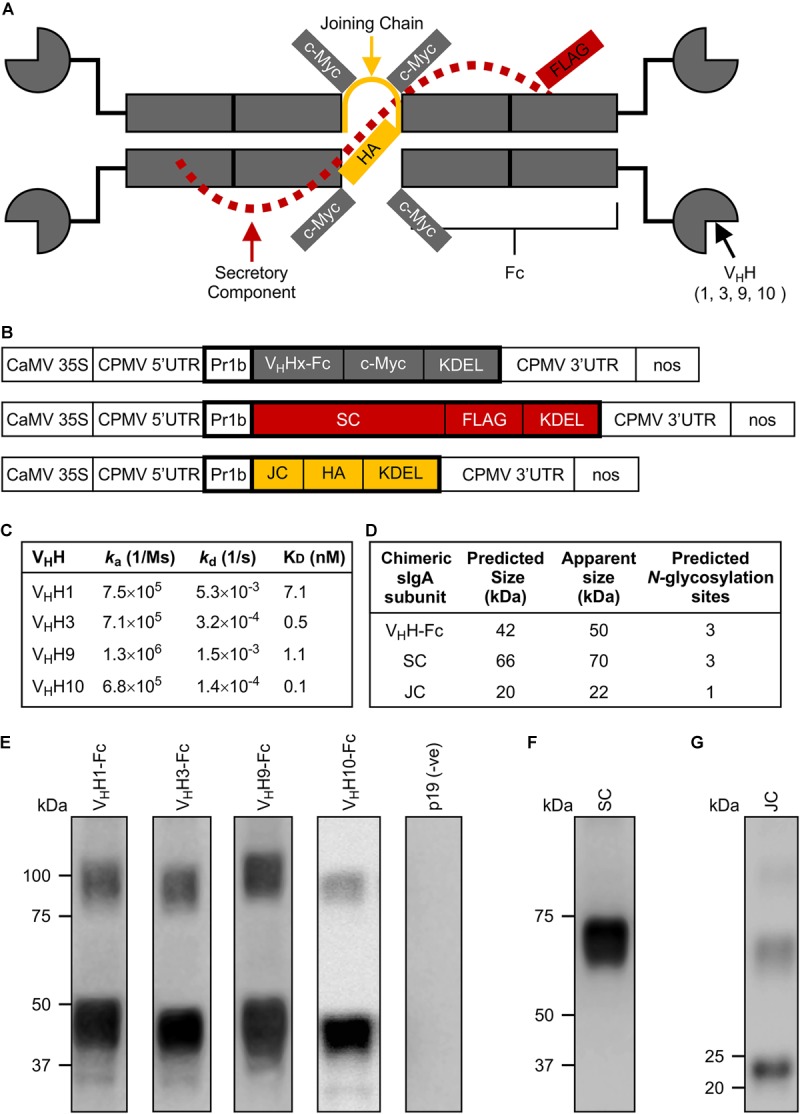
Design and production of individual subunits required for chimeric secretory IgA assembly. **(A)** Schematic of all produced subunits fully assembled into a chimeric antibody intended for secretory IgA functionality. It notably differs from the structure of native secretory IgA by the replacement of the Fab region with a camelid-derived variable heavy chain fragment (V_H_H). **(B)** Schematic representation of constructs used for *Agrobacterium*-mediated transient expression in *N. benthamiana* leaves. CaMV 35S, cauliflower mosaic virus 35S promoter; CPMV 5′UTR, 5′-untranslated region of Cowpea mosaic virus; PR1b, tobacco pathogenesis-related protein 1b signal peptide; V_H_Hx-Fc, fusion of a camelid-derived V_H_H to a bovine Fc where x is either 1, 3, 9, or 10, corresponding to the isolated V_H_Hs; SC, bovine secretory component; JC, bovine JC; c-Myc, FLAG, HA, detection tags; KDEL, endoplasmic reticulum retrieval tetra-peptide; CPMV 3′UTR, 3′-untranslated region of Cowpea mosaic virus; nos, nopaline synthase terminator sequence; the cassettes were cloned into pEAQ-DEST-1 plant expression vectors. Schematic not drawn to scale. Bold outlines indicate translated regions. **(C)** Monovalent affinities and kinetics of the interaction between V_H_Hs and MBP-Int277 by SPR (pH 7.4, 25°C). **(D)** Predicted protein size and number of glycosylation sites for each subunit. **(E–G)** Western blots of crude extract from leaves of *N. benthamiana* harvested at 6 dpi expressing V_H_H1, 3, 9, and 10-Fc along with p19, a suppressor of gene silencing **(E)**, SC **(F)**, and JC **(G)**. 10 μg of TSP was loaded in each lane.

With the intent of blocking the interaction of EHEC with the intestinal mucosa, we immunized a llama with the C-terminal 277 residues of intimin, which extend extracellularly from the bacterial cell and mediate interaction with intestinal epithelial cells via binding to its cognate translocated receptor (Frankel et al., 1994). We produced and panned a phage-displayed library of llama heavy chain only antibody variable domains (V_H_Hs) and identified V_H_Hs that could bind and neutralize intimin γ, the main subtype associated with O157:H7. With passive mucosal immunotherapy and diagnostic development as end goals, we developed a chimeric antibody by fusing each of the isolated V_H_Hs to a bovine IgA Fc and co-expressing them with both JC and SC subunits to enable sIgA functionality. Unlike native mammalian sIgA which consists of four light chains, four heavy chains, one JC, and one SC, the chimeric antibody (V_H_H-sIgA) is composed of four V_H_H-Fc heavy chain-only subunits, one SC, and one JC (Figure 1A). We demonstrated the recombinant production and correct assembly of the chimeric V_H_H-sIgA against intimin γ in *Nicotiana benthamiana*. We also characterized its binding to and neutralization of the O157:H7 serotype as well as the “Big Six” serotypes. This study is notable because of the potential for development of an oral passive mucosal immunotherapeutic capable of multivalent protection, and as a diagnostic tool for detection of four of the seven most prevalent EHEC strains.

## Results

### Camelid V_H_Hs Recognize EHEC O157:H7 Intimin With High Affinity

We selected the C-terminal 277 residues of intimin (Int-277) as the intended V_H_H-sIgA target because this region has previously been demonstrated to be immunogenic and to elicit IgAs (McKee and O’Brien, 1996; Gansheroff et al., 1999). Int-277 of EHEC O157:H7 strain EDL933 intimin γ was fused to maltose-binding protein (MBP) and the resulting fusion (MBP-Int277) was expressed in *E. coli* BL21 (DE3) cells by GenScript USA, Inc. (NJ, United States; Supplementary Figure S1A). Characterization of the MBP-Int277 protein by SDS-PAGE, western blotting, size exclusion chromatography, and ELISA using an anti-intimin antibody (Carvalho et al., 2005) collectively suggested that MBP-Int277 had the expected size (73.8 kDa), was monodisperse, and was correctly folded (Supplementary Figures S1B–E). Because camelids can generate heavy chain-only antibodies, a llama was immunized with MBP-Int277. A competition ELISA using a polyclonal anti-intimin antibody (Carvalho et al., 2005) suggested that the polyclonal antibody response in the llama was directed substantially toward Int277 rather than MBP, based on almost complete knock down of the anti-intimin antibody in the presence of serum from the immunized llama (Supplementary Figure S1E). A phage-displayed V_H_H library was produced and panned for intimin-specific V_H_H sequences. Of five V_H_Hs, four (V_H_H1, 3, 9, and 10) showed low-nanomolar monovalent binding affinities for intimin based on surface plasmon resonance (SPR) and had no binding to MBP alone (Figure 1C).

### Production of Chimeric sIgA Subunits in *N. benthamiana*

To produce chimeric V_H_H-IgAs, each V_H_H sequence was fused to a bovine IgA Fc (V_H_H-Fc). Each of the V_H_H-Fc, SC, and JC subunits were fused to the PR1b signal peptide and KDEL retrieval signal peptide to enable ER targeting and localization, as well as c-Myc, FLAG, and HA tags, respectively, to enable separate detection of the subunits upon co-expression (Figure 1A,B). For production in plant leaf tissue, the constructs were codon-optimized for *N. benthamiana* nuclear expression, cloned separately into pEAQ-DEST-1 (Sainsbury et al., 2009) plant expression vectors, and verified by DNA sequencing. *Agrobacterium tumefaciens* was transformed with each construct, then *N. benthamiana* leaves were co-infiltrated with each *Agrobacterium* strain along with an *Agrobacterium* strain containing p19, a suppressor of gene silencing from *Cymbidium* ringspot virus (CymRSV) (Silhavy et al., 2002; Saberianfar et al., 2015). Accumulation of each subunit at 6 days post-infiltration (dpi) was evaluated by western blotting using either anti-c-myc, anti-FLAG, or anti-HA antibodies to detect the respective subunits (Figure 1E–G). All subunits appeared to be of slightly higher molecular mass than their respective predicted molecular weights based on amino acid residues only, presumably due to glycosylation (Figure 1D). In the fully assembled native sIgA complex, each of the V_H_H-Fc, SC, and JC chains are predicted to have three, three, and one *N*-glycosylation sites, respectively (Steentoft et al., 2013). The glycans on native sIgA have been shown to protect the structure from proteolytic degradation in the harsh mucosal environment and may also exhibit some neutralization capacity against some bacterial strains by sterically hindering attachment of sugar-dependent receptors or fimbriae to epithelial cells (Wold et al., 1990; Ruhl et al., 1996; Royle et al., 2003).

### Optimizing Co-expression of Chimeric sIgA Subunits

**FIGURE 2:**
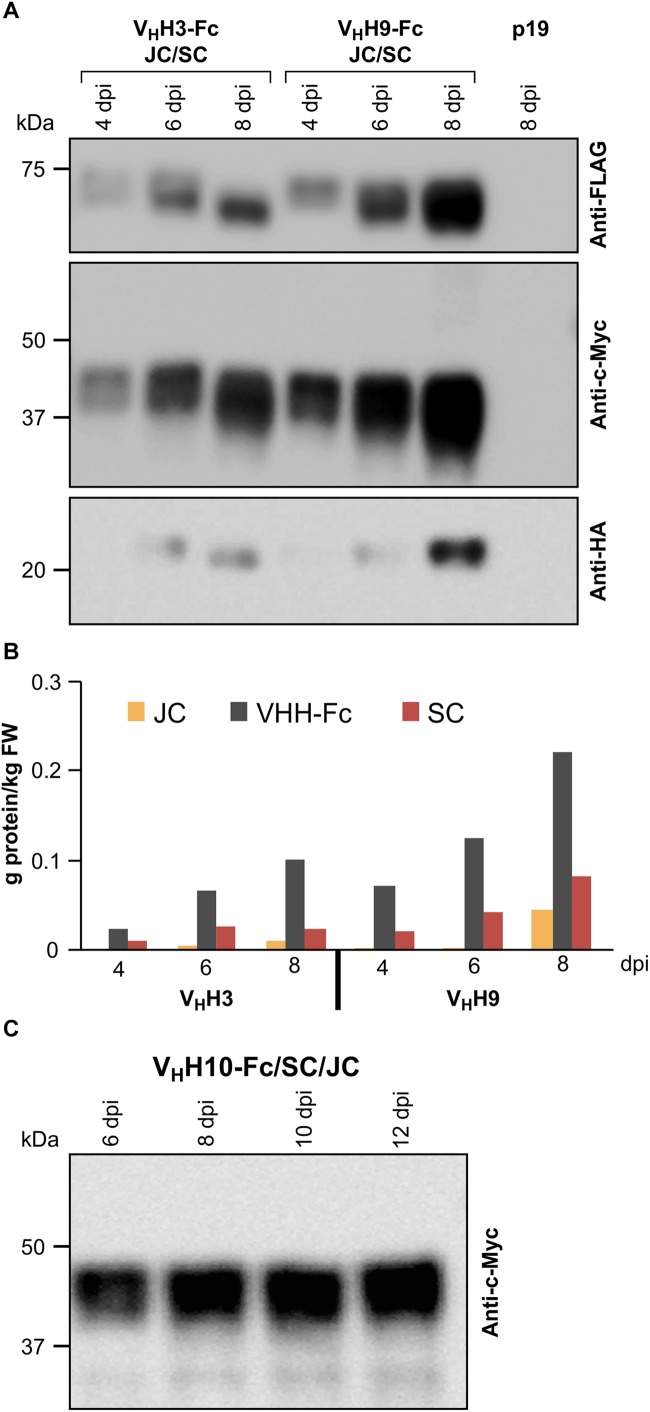
Optimization of the accumulation levels of V_H_H-sIgA subunits after transient transformation of *N. benthamiana* leaves. **(A)** *N. benthamiana* leaf tissue was infiltrated with *Agrobacterium* mixtures containing the following ODs (*A*_600_); V_H_H-Fc: 0.57, SC: 0.14, JC: 0.14, and p19: 0.14. Leaf tissue was collected 4–8 dpi. TSP was extracted from pooled samples from three independent biological replicates. Ten micrograms of TSP was loaded in each well, separated by SDS-PAGE under reducing conditions, and visualized by western blot. Known amounts of c-Myc, HA, and FLAG tagged protein were used as reference (not shown). TSP from p19-infiltrated *N. benthamiana* leaves was used as a negative control. **(B)** Quantification of **(A)** by densitometry. **(C)** Time-course (6–12 dpi) of V_H_H10-Fc accumulation in combination with SC and JC.

The correct assembly of the chimeric sIgA into a hetero-multimeric protein complex likely requires the nascent polypeptides to be temporally and spatially coordinated in a predicted 4:1:1 stoichiometric ratio of V_H_H-Fc:SC:JC. To optimize the conditions for producing the assembled complex, we tested a range of *Agrobacterium* ratios for co-infiltration in *N. benthamiana* leaves (VHH-Fc:SC:JC of 1:1:1, 4:1:1, 4:1:2). We obtained accumulation levels of the subunits (g/kg) closest to the 4:1:1 present in the assembled sIgA with an *Agrobacterium* ratio of 4:1:2. Infiltration cultures were prepared by mixing *Agrobacterium* strains containing V_H_H3-Fc or V_H_H9-Fc with *Agrobacterium* strains containing SC, JC, and p19 at optical densities (OD at *A*_600_) of 0.50, 0.12, 0.24, and 0.12, respectively. The accumulation levels of each subunit were measured from 4 to 8 dpi. Accumulation levels of all three subunits in both infiltration mixtures peaked at 8 dpi (Figure 2A,B). V_H_H9-Fc mixtures reached the highest accumulation levels for all three subunits with V_H_H9-Fc at 0.22 g/kg, SC at 0.08 g/kg, and JC at 0.04 g/kg, resulting in a total of approximately 0.34 g/kg for sIgA subunits, which when converted to molar ratios result in 4.2:1:1.6 (V_H_H9-Fc:SC:JC). This combination was the closest to the expected 4:1:1 molar ratio required for assembly of sIgA and should allow for *in vivo* assembly of the subunits into a hetero-multimeric protein complex.

Since the highest accumulation levels for V_H_H3-sIgA and V_H_H9-sIgA were reached at 8 dpi, the accumulation levels of V_H_H10-sIgA were monitored beyond 8 dpi to examine if higher accumulation could be achieved. We used similar *Agrobacterium* ODs for infiltration mixtures (0.57:0.14:0.14:0.14) as in the previous experiment, and monitored the accumulation of V_H_H10-Fc up to 12 dpi (Figure 2C). Over the course of the experiment, V_H_H10-Fc accumulated well up to 12 dpi. The accumulation of V_H_H10-Fc reached 0.12 g/kg fresh weight (FW).

### The Chimeric sIgA Subunits Associate *in vivo*

**FIGURE 3:**
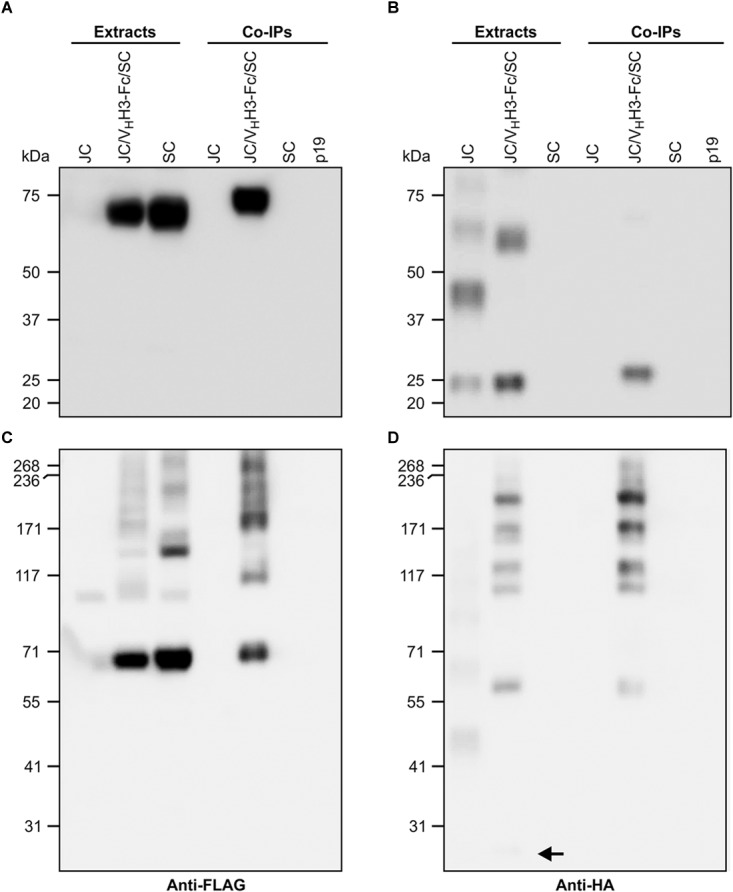
V_H_H3-sIgA subunits physically associate with one another. *N. benthamiana* leaf tissue co-infiltrated with all V_H_H3-sIgA subunits was collected at 6 dpi, and recombinant proteins were immunoprecipitated with an anti-c-Myc antibody. **(A,B)** Both cell extracts and immunoprecipitates were resolved by SDS-PAGE under reducing conditions. **(C,D)** SDS-PAGE performed under non-reducing conditions. **(A,C)** Immunoblots were detected with anti-FLAG antibodies. **(B,D)** Immunoblots were detected with anti-HA antibodies. Arrow points to a faint but nonetheless present monomeric JC band. TSP from p19-infiltrated *N. benthamiana* leaves was used as negative control.

Subunits of native sIgA are known to be covalently linked by disulfide bonds. To determine if the co-expressed subunits were physically associating, crude extracts of leaves infiltrated with V_H_H3-Fc/SC/JC were immunoprecipitated with the c-Myc antibody specific to the V_H_H-Fc subunit. The immunoprecipitated proteins were detected on a western blot with either anti-FLAG antibody specific to the SC subunit (Figure 3A,C) or anti-HA antibody specific to the JC subunit (Figure 3B,D). When the proteins were separated under reducing PAGE conditions, the ∼70-kDa SC subunit was detected in the extracts containing SC only and those containing all three subunits. However, after co-immunoprecipitation (co-IP), SC was only detected in the treatment containing all three subunits (Figure 3A), indicating that it was associated with the V_H_H-Fc subunit. Similarly, the ∼20-kDa JC was detected in extracts containing JC only and those containing all three subunits, but after co-IP, JC was only detected in the treatment containing all three subunits (Figure 3B). When the same samples were separated by non-reducing PAGE, SC expressed alone appeared as a main band at 70-kDa, with a ladder of larger products presumably representing multimerization via non-specific disulfide bond formation. When all three subunits were present in the cell extract (V_H_H3-Fc/SC/JC), several other intermediate products were detected. After co-IP, bands were only observed in extracts containing all three subunits, including a band running around 250 kDa, the expected size of the fully assembled chimeric sIgA (Figure 3C). Similarly, upon detection with anti-HA, we observed several faint bands representing JC multimers, and in the V_H_H3-Fc/SC/JC lane, JC monomer (shown with an arrow) and several other intermediate products were detected. As expected, after co-IP and detection with anti-HA, the same bands were only observed in the treatment containing all three subunits (Figure 3D). Co-IP experiments were performed with all constructs and similar results were consistently observed in every case (data not shown).

### Secretory IgA Subunits Assemble Into a Hetero-Multimeric Protein Complex *in vivo*

**FIGURE 4:**
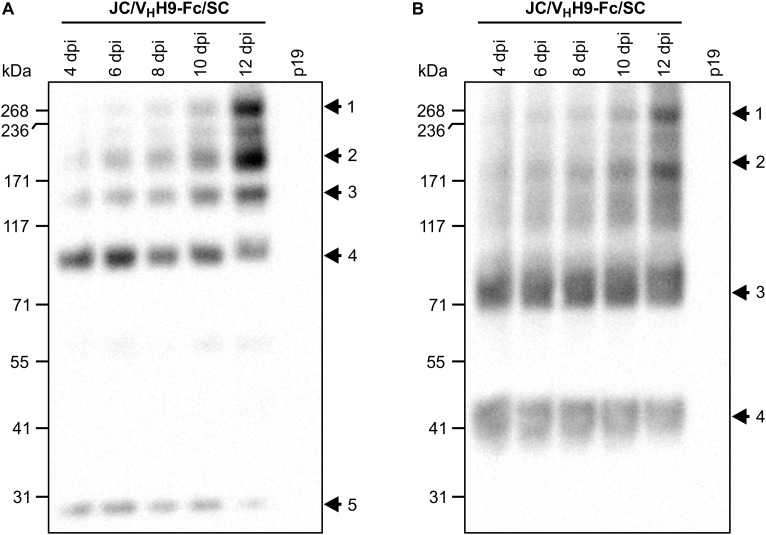
V_H_H9-sIgA subunits assemble *in vivo* over time. *N. benthamiana* leaves were co-infiltrated with a mixture of the indicated *Agrobacterium* strains, and leaf samples were collected from 4 to 12 dpi. Extracts were prepared under native conditions and separated with SDS-PAGE under non-reducing conditions. Extract from p19-infiltrated *N. benthamiana* leaves at 12 dpi was used as negative control. Equal amounts (10 μg) of TSP were loaded per lane. Western blot was detected with anti-HA **(A)** and anti-c-Myc antibodies **(B)**.

Although we determined that V_H_H-Fc accumulation peaks at 8 dpi, it was not clear how fast the subunits assemble into the chimeric sIgA complex. Therefore, a time-course experiment was performed in which leaf tissue was collected every 2 days from 4 to 12 dpi, separated by SDS-PAGE under non-reducing conditions, and detected with anti-HA (Figure 4A), anti-c-Myc (Figure 4B), and anti-FLAG (data not shown) antibodies. The results indicated that assembly of the chimeric sIgA and intermediates was gradual and continued through 12 dpi (Figure 4A, arrows 1–3; Figure 4B, arrows 1–2), while monomeric JC and a 90-kDa intermediate (Figure 4A, arrows 4–5) and monomeric V_H_H9-Fc and an 80-kDa intermediate (Figure 4B, arrows 3–4) showed diminishing accumulation across the same period. Taken together, these data suggest that the chimeric sIgA assembled with time, and that a later harvest may be beneficial.

### Vacuum Infiltration and Purification of V_H_H9-sIgA

**FIGURE 5:**
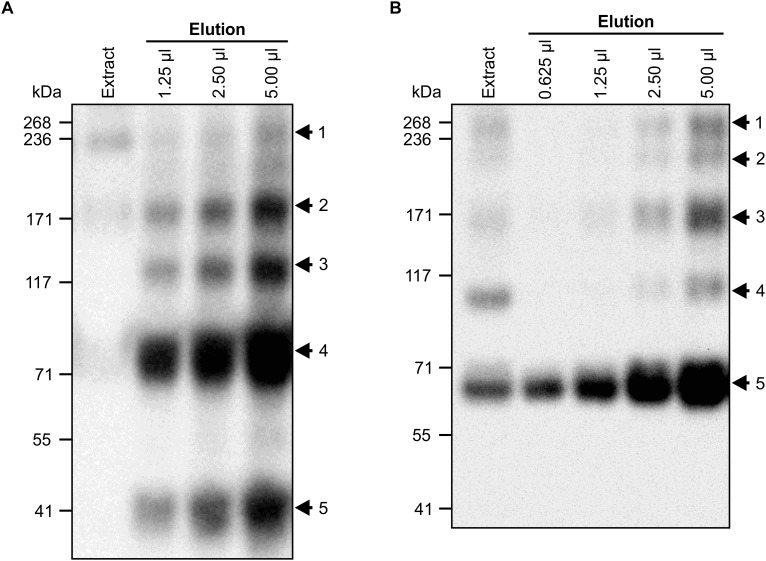
Vacuum infiltration and purification of V_H_H9-sIgA. *N. benthamiana* leaves were vacuum infiltrated with a mixture of V_H_H9-Fc/SC/JC and p19. Tissue was collected at 12 dpi. Cell extracts were prepared under native conditions and separated with SDS-PAGE under non-reducing conditions. **(A)** Secretory IgA was purified with peptide M Agarose. Western blots were detected with anti-c-Myc antibody. Arrows indicate the expected size of fully assembled sIgA (No. 1), tetrameric (No. 2, ∼176 kDa), trimeric (No. 3, ∼132 kDa), dimeric (No. 4, ∼88 kDa), and monomeric (No. 5, ∼44 kDa) V_H_H9-Fc. **(B)** Secretory IgA was purified with anti-FLAG agarose. Western blots were detected with anti-Flag antibody. Arrows indicate the expected size of fully assembled sIgA (No. 1, ∼66 kDa), SC/trimeric V_H_H9-Fc/JC (No. 2, ∼206 kDa), SC/dimeric V_H_H9-Fc (No. 3, ∼160), SC/monomeric V_H_H9-Fc (No. 4, ∼110 kDa), and monomeric SC (No. 5). 10 μl of cell extract was loaded as a snapshot of the antibody produced *in vivo*.

To characterize the antigen- and pathogen-binding of chimeric V_H_H9-sIgA, large quantities of the purified assembled protein complex were required. Therefore, *N. benthamiana* plants were vacuum-infiltrated, and leaves were collected at 12 dpi. While IgG is routinely purified using protein A or protein G resins, there are no efficient methodologies available for purifying IgA molecules lacking light chains. Therefore, we compared two methods for purifying V_H_H9-sIgA. The first method took advantage of a peptide derived from a surface protein of *Streptococcus pyogenes*, peptide M, which binds to the Fc region of bovine IgA. The second purification method used an affinity resin that binds the FLAG tag we fused to SC (Figure 5).

In the crude leaf extract, V_H_H9-sIgA was the main product observed on a western blot detected with the c-Myc antibody (Figure 5A, extract lane, arrow 1). When purified with peptide M, unassembled and partially multimerized V_H_H9-Fc polypeptides were heavily enriched, and several bands were observed (Figure 5A). The strongest bands belonged to monomeric (∼44 kDa) and dimeric (∼88 kDa) V_H_H9-Fc (Figure 5A, arrows 4 and 5). In addition, three other bands were observed that correspond to the trimeric (∼132 kDa) and tetrameric (∼176 kDa) V_H_H9-Fc, and a fainter band representing the fully assembled chimeric sIgA (∼270 kDa) (Figure 5A, bands 3, 2, and 1, respectively). This method of purification was efficient and allowed recovery of 0.6 mg/ml of c-Myc-reactive antibody fragments, as estimated by whole lane densitometry against known amounts of a standard protein. However, purification with peptide M preferentially recovered monomeric and dimeric V_H_H9-Fc compared with fully assembled V_H_H9-sIgA.

We also used anti-FLAG agarose for purification of V_H_H9-sIgA in a second attempt to enrich for the fully assembled chimeric sIgA. We hypothesized that this method of purification should allow a higher recovery of the fully assembled sIgA since the FLAG tag is located on the SC subunit which is wrapped around the fully assembled sIgA complex. After Western blot and detection with anti-FLAG antibody, we observed several bands. The strongest band belonged to free or monomeric SC (∼66 kDa) (Figure 5B, arrow 5), but the fully assembled V_H_H9-sIgA band was much more prominent than following peptide M purification (∼270 kDa) (Figure 5B, arrow 1). Three other bands were also recovered which we speculate belong to sIgA intermediate products such as SC/trimeric V_H_H9-Fc/JC (∼206 kDa), SC/dimeric V_H_H9-Fc (∼160 kDa), and SC/V_H_H9-Fc (No. 4, ∼110 kDa) (Figure 5B, bands 4, 3, and 2, respectively). However, whole lane densitometry indicated that the purification with anti-FLAG agarose recovered much less protein (0.014 mg/ml) than peptide M (0.6 mg/ml).

### Plant-Produced V_H_H9-sIgA Is Antigen-Binding Competent

**FIGURE 6:**
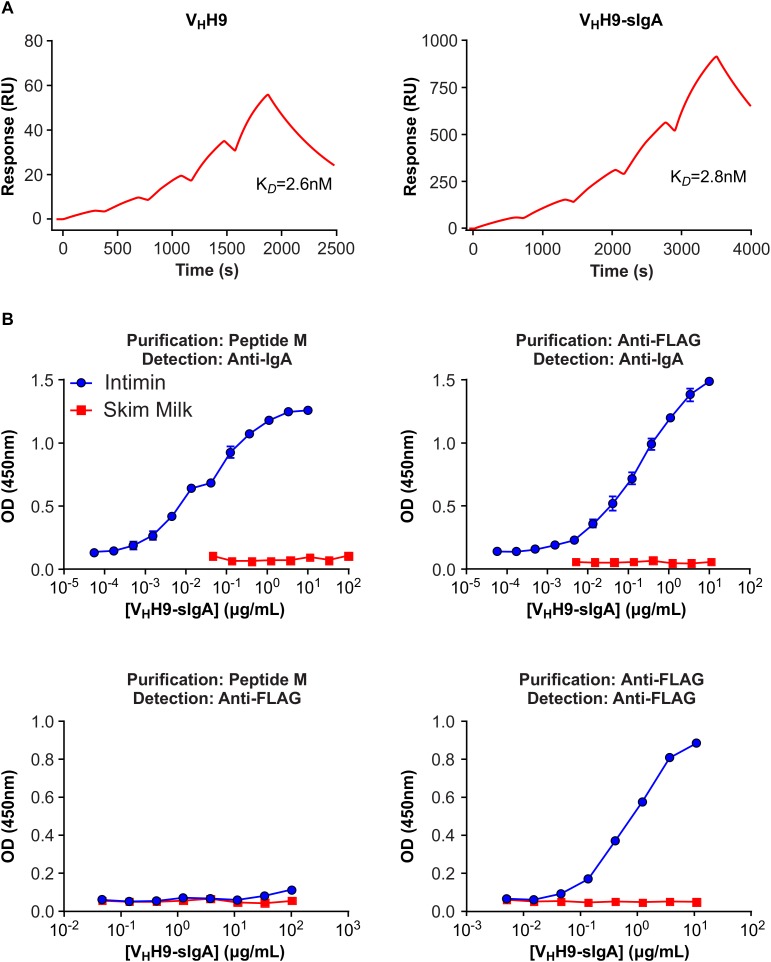
Binding of plant-produced V_H_H9-sIgA to EHEC O157:H7 intimin. **(A)** SPR binding of V_H_H9-sIgA purified using peptide M. Either plant-produced V_H_H9-sIgA (top) or *E. coli*-produced V_H_H9 monomer (bottom) was immobilized on CM5 Series S sensor chips via amine coupling and MBP-Int277 was flowed over the resulting surfaces at concentrations ranging from 0.3 to 5 nM. The experiment was conducted in duplicate. Black lines show data and red lines show fits. **(B)** ELISA binding of plant-produced V_H_H9-sIgA purified using either peptide M (left) or anti-FLAG antibody (right) and detected using either anti-bovine IgA antibody (top) or anti-FLAG antibody (bottom). Results are representative of two independent experiments.

Intimin binding by plant-produced V_H_H9-Fc purified either using peptide M (yielding all V_H_H9-Fc molecules regardless of the presence of JC and SC) or anti-FLAG antibody (yielding SC as well as secretory V_H_H9-Fc in complex with SC, and possibly JC) was assessed by SPR and ELISA. No loss of intimin-binding affinity was observed by SPR for peptide M-purified V_H_H9-Fc produced *in planta* compared with V_H_H9 monomer produced in *E. coli* (Figure 6A). Moreover, both peptide M-purified and anti-FLAG-purified V_H_H9-Fc bound intimin with similar half maximal effective concentrations (EC50s) in ELISAs detected with horseradish peroxidase (HRP)-conjugated anti-bovine IgG antibody (Figure 6B). However, no binding of peptide M-purified V_H_H9-Fc was observed in ELISAs detected with anti-FLAG antibody, suggesting that little SC was present in the purified material.

### Plant-Produced V_H_H10-sIgA Binds EHEC Strains O26:H11, O145:Hnm, O111:Hnm, and O157:H7

**FIGURE 7:**
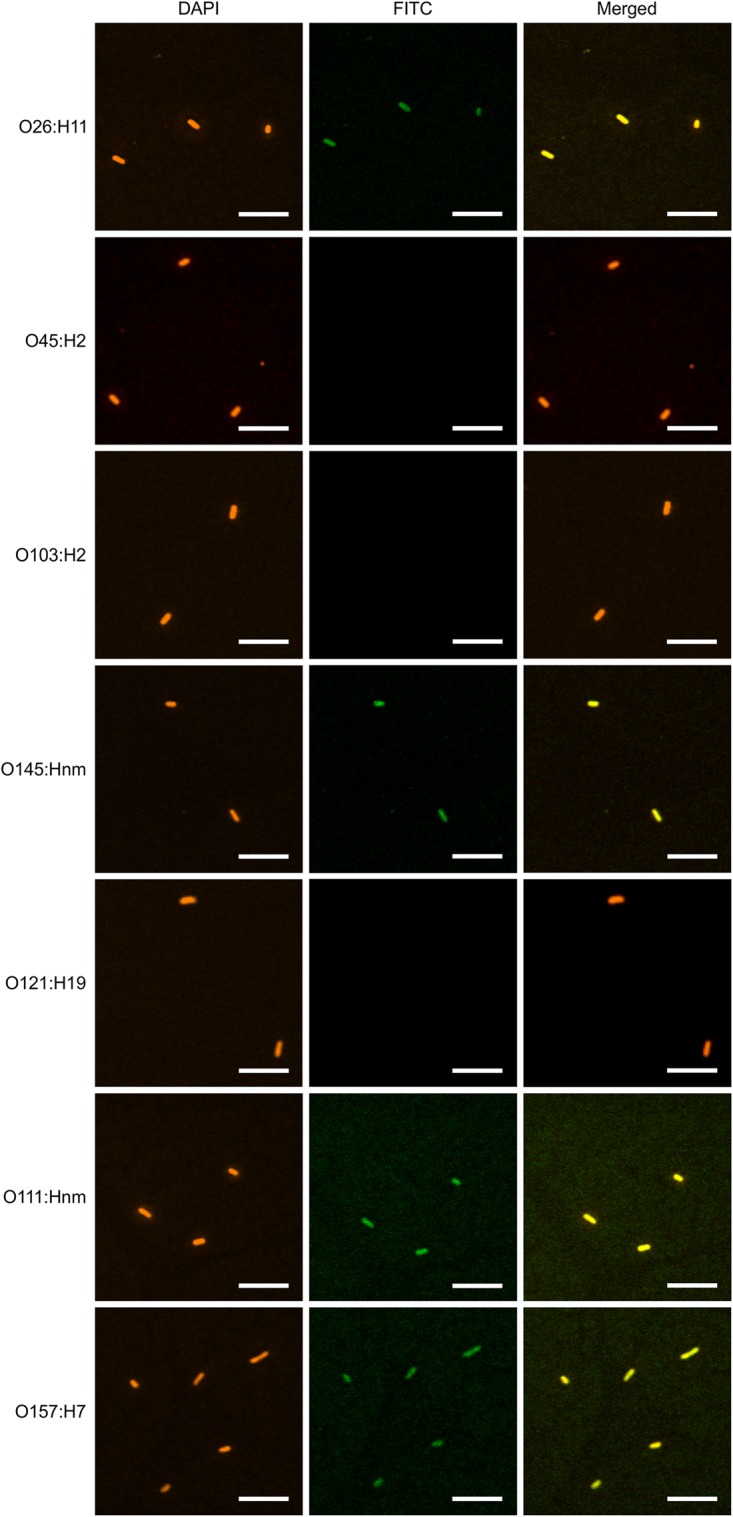
V_H_H10-sIgA binds EHEC O145:Hnm, O26:H11, O111:Hnm, and O157:H7. Shown are confocal images visualizing the binding of the seven most prevalent strains of EHEC with V_H_H10-sIgA. Binding is visualized by DAPI that stains EHEC bacterial cells (blue) and a FITC-conjugated antibody that hybridizes to the Fc chain of V_H_H10-sIgA (green). A merged image shows an overlay of the blue and green channels used to visualize DAPI and FITC, respectively, to assess co-localization.

**FIGURE 8:**
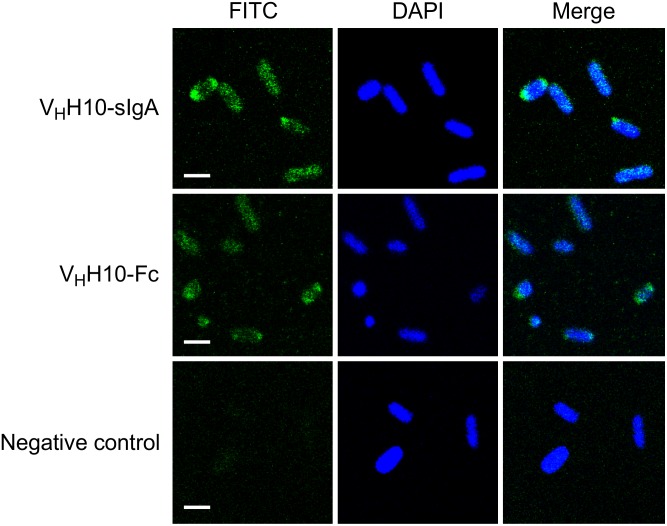
Plant-produced V_H_H10-Fc chain binds to *E. coli* O157:H7 by itself as well as when co-expressed with the JC and SC as a sIgA complex. Shown are confocal images visualizing co-localization of a FITC-conjugated antibody that hybridizes to the Fc chain of either V_H_H10-sIgA (V_H_H10-Fc/SC/JC) or V_H_H10-Fc as well as DAPI that stains *E. coli* O157:H7 bacterial cells. *E. coli* cells treated with PBS-T instead of antibody were used as a negative control. Merge image represents an overlay of signals from both green and blue channels used for localization of antibodies and *E. coli* cells, respectively. Bar, 2 μm.

To determine if plant-produced chimeric V_H_H-sIgA antibodies bind to the seven most prevalent strains of EHEC, bacterial cells of O26:H11, O45:H2, O103:H2, O145:Hnm, O121:H19, O111:Hnm, and O157:H7 were incubated with V_H_H10-sIgA purified using anti-FLAG (binds the SC), then visualized using a secondary fluorescent antibody (rabbit anti-bovine-FITC) that binds the Fc and 4′,6-diaminodino-2-phenylindole (DAPI) that stains bacterial cells. The confocal images showed consistent co-localization of FITC signal with strains O26:H11, O145:Hnm, O111:Hnm, and O157:H7 cells (Figure 7). Since the heavily glycosylated SC has been reported to interact with some bacterial strains, we were concerned that the observed co-localization could be a product of non-specific glycan-mediated binding, and not binding of the V_H_H to intimin. To address this, we compared binding of VHH10-sIgA with binding of V_H_H10-Fc expressed alone to EHEC O157:H7. Confocal images showed co-localization of V_H_H10-Fc with EHEC O157:H7 cells in the absence of SC and JC, suggesting that binding was likely V_H_H-mediated (Figure 8). As a negative control, EHEC cells were also treated with PBS containing 0.1% Tween-20 (PBS-T) instead of antibodies and similarly stained but did not show fluorescence under FITC-related imaging conditions (480 nm excitation and 520–540 nm detection) (Figure 8).

### Plant-Produced V_H_H10-sIgA Reduces Adherence of Three EHEC Serotypes to Epithelial Cells

**FIGURE 9:**
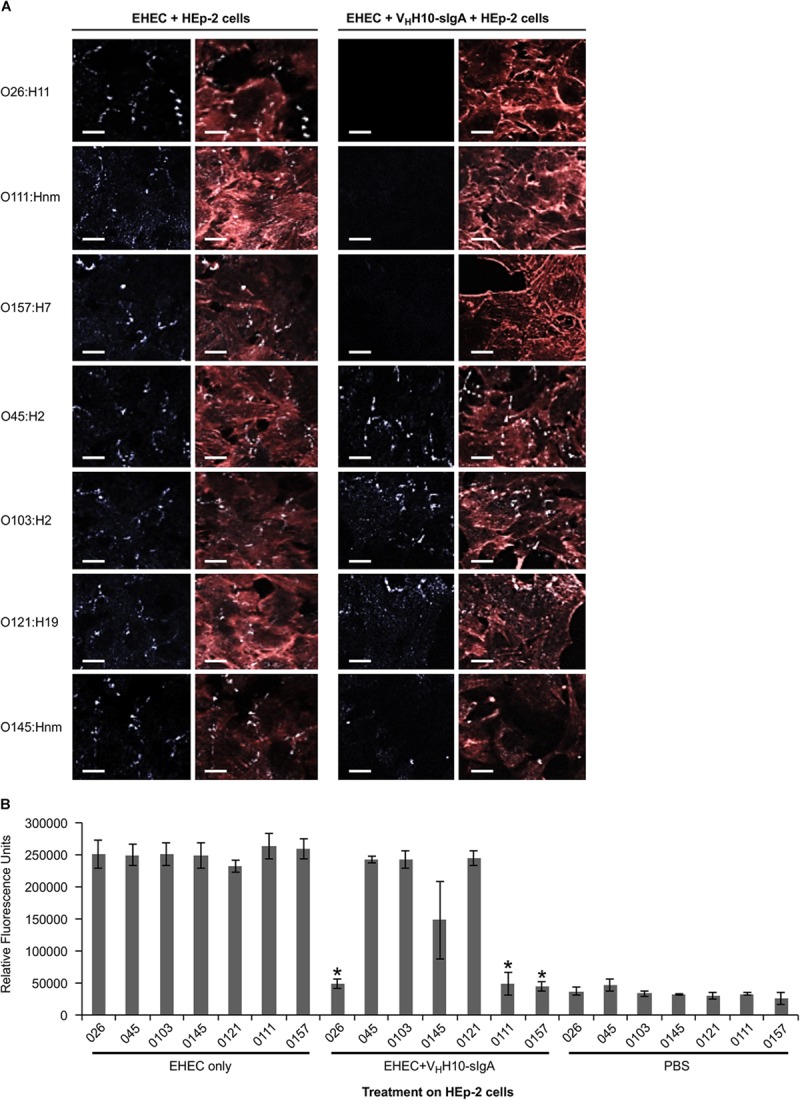
V_H_H10-sIgA neutralizes EHEC O26:H11, O111:Hnm, and O157:H7 adhesion to HEp-2 cells. **(A)** Neutralization was visualized by immunolabelling EHEC cells with a donkey anti-rabbit secondary antibody (white) as well as the actin cytoskeleton of HEp-2 cells using rhodamine phalloidin (red). Shown are merged images of the red and white channels for either HEp-2 cells incubated with EHEC alone (left panel) or with EHEC and V_H_H10-sIgA (right panel). **(B)** V_H_H10-sIgA reduces fluorescence of O26:H11, O111:Hnm, and O157:H7 to background levels. Shown is the relative fluorescence of EHEC strains that have been immunolabeled, are adherent on HEp-2 cells, and either incubated on HEp-2 cells alone or in combination with V_H_H10-sIgA. As a negative control, HEp-2 cells were incubated with PBS instead of a bacterial strain or antibody. ^∗^ indicates a significant reduction of the amount of immunolabeled adherent bacteria as determined by a one-tailed unpaired homoscedastic *T*-test between an EHEC strain alone versus the same EHEC strain with V_H_H10-sIgA added (*p* < 0.05, *N* = 3 biological replicates). Error bars indicate standard errors of the means.

Since intimin mediates the intimate attachment of EHEC to epithelial cells, we investigated if the binding of V_H_H10-sIgA to EHEC could neutralize the ability of bacteria to adhere to epithelial cells. HEp-2 cells were incubated with a culture of one of seven EHEC strains (O26:H11, O45:H2, O103:H2, O145:Hnm, O121:H19, O111:Hnm, and O157:H7) in the presence or absence of V_H_H10-sIgA, washed to remove any non-adherent bacteria, and then visualized by immunofluorescence microscopy. HEp-2 cells were visualized by fluorescent actin staining using rhodamine phalloidin (red) and EHEC cells using a donkey anti-rabbit Alexa 350-conjugated secondary antibody (shown in white). Compared to the respective positive controls of HEp-2 cells and EHEC only, the addition of V_H_H10-sIgA seemed to abrogate the adhesion of EHEC strains O26:H11, O111:Hnm, and O157:H7 to HEp-2 cells, while it seems to somewhat reduce adhesion of EHEC strain O145:Hnm to HEp-2 cells (Figure 9A). To quantify the neutralization capacity of V_H_H10-sIgA, we adapted the adhesion assay for fluorometry and measured the relative fluorescence of HEp-2 cells incubated with a culture of each of the seven EHEC strains with and without V_H_H10-sIgA. The addition of V_H_H10-sIgA afforded complete protection, that is, it reduced the relative fluorescence caused by adherent bacteria for strains O26:H11, O111:Hnm, and O157:H7 to background levels, and somewhat reduced the relative fluorescence caused by adherent bacteria for strain O145:nm, although this effect was not statistically significant (*p* = 0.09 in a *T*-test; Figure 9B).

We performed a multiple sequence alignment and derived a neighbor-joining tree of Int277 across all seven strains and found that EHEC strains O157, O111, O145, and O26 grouped together based on sequence similarity, while O45, O103, and O121 were more disparate in sequence (Supplementary Figure S2). With the exception of O145, this is in accord with V_H_H10-sIgA being able to bind and neutralize O26, O111, and O157 but not bind and neutralize O45, O103, and O121.

## Discussion

### Critical Factors Involved in Chimeric V_H_H-sIgA Production: High Accumulation Levels and Optimal Stoichiometric Ratio of the Subunits

Chimeric V_H_H-sIgA is a complex molecule composed of six subunits that require assembly into a functional unit. To achieve this, three key goals should be met: high accumulation levels of individual subunits, optimal molar ratio of the subunits, and proper assembly of the subunits into the multimeric complex.

To ensure high accumulation levels, proper folding, and post-translational modifications of recombinant proteins, we targeted the proteins of interest to the secretory pathway and retrieved them to the ER with an ER-retrieval tetrapeptide signal (KDEL) (Saberianfar et al., 2015; Xu and Ng, 2015; Saberianfar and Menassa, 2017).

Several strategies have been suggested to reach the optimal ratio for assembly of the sIgA subunits such as using promoters with varying strengths in multi-cassette vectors, or even *in vitro* re-association (or reconstitution) of the sIgA subunits (Longet et al., 2014). In practice however, empirical determination is necessary (Virdi et al., 2016). To ensure the desired 4:1:1 stoichiometric ratio of V_H_H-Fc:SC:JC that we hypothesized would be optimal for assembly and accumulation of this chimeric sIgA, we used different amounts of *Agrobacterium* for co-infiltrations of *N. benthamiana* leaves, and achieved relatively high accumulation levels up to 0.34 mg/g FW (3.24% of total soluble protein, TSP).

### Time and Subunit Assembly as Limiting Factors for sIgA Production

When each of the sIgA subunits was transiently expressed alone, the V_H_H-Fc and SC subunits accumulated to relatively high levels, whereas the JC accumulated to much lower levels (Figure 1E–G). Higher accumulation of the JC was detected after co-expression with the V_H_H-Fc and SC subunits (Figure 2A,B), and both recombinant protein accumulation levels and sIgA assembly increased over time (Figure 2, 4). Collectively, this suggests that upon co-expression, the chimeric sIgA subunits assemble *in vivo* over time into a complex that is more stable to degradation than the nascent unassembled chains, particularly with regards to the JC. Indeed, the incorporation of the JC into the dimeric IgA complex has previously been reported to be a key limiting factor for sIgA production *in planta* (Westerhof et al., 2015). Our findings support this, and furthermore suggest that stabilizing the JC may be a key target for further optimization of sIgA production.

### Plant-Produced V_H_H-sIgA Binds *E. coli* O26:H11, O145:Hnm, O111:Hnm, and O157:H7

We showed by SPR that both the plant-produced V_H_H9-Fc chain as well as the assembled V_H_H9-sIgA complex have the same binding affinity as monomeric V_H_H9 produced in *E. coli*, suggesting that binding is modularly mediated and retained via the V_H_H following Fc fusion and assembly with the SC and JC. Because V_H_H10 was shown to have superior binding affinity by SPR, it was chosen to be advanced toward pathogen binding and neutralization assays. We observed consistent co-localization of V_H_H10-sIgA with strains O26:H11, O145:Hnm, O111:Hnm, and O157:H7 cells by immunofluorescence confocal microscopy. Coverage was observed across the entirety of the cells unlike previous reports of partial binding for rat- and chicken-produced antibodies against EHEC (Cook et al., 2007), suggesting that intimin is abundantly embedded across the entire cell surface membrane of these four strains and is accessible to V_H_H10-sIgA.

### Plant-Produced V_H_H10-sIgA Neutralizes the Ability of EHEC Strains O26:H11, O111:Hnm, and O157:H7 to Adhere to Epithelial Cells

All EHEC strains use a highly conserved type III secretion system to enable colonization of intestinal epithelial cells. Intimate adherence mediated by intimin docking to its translocated cognate receptor is a necessary prerequisite for invasion and virulence (Dziva et al., 2004; Buttner, 2012). Given that our results indicate that V_H_H10-sIgA prevents intimate adherence for strains O26:H11, O111:Hnm, and O157:H7, it is tempting to speculate that this protective effect will also be observed when used in animal trials. Although V_H_H10-sIgA was able to bind O145:Hnm, fluorometry and confocal images of the adhesion assay suggested a compromised ability to neutralize. It is possible that V_H_H10-sIgA can partially bind O145:Hnm but not sufficiently to prevent intimate adherence to epithelial cells. Regarding the differential capacity of V_H_H10-sIgA to bind and neutralize across strains, we speculate that this may be due to sequence variability across the C-terminal 277 residues of the intimin protein. Although the transmembrane and intracellular residues are strongly conserved in native intimin, the extracellular Int277 region is highly variable and is likely shaped by selection pressures of the host immune system. Despite O145 being similar in sequence to O157, weaker binding and neutralization of O145 may be due to sequence variability at a local epitope to which V_H_H10 binds rather than sequence conservation of Int277 as a whole.

The finding that V_H_H10-sIgA offers multivalent protection against EHEC O26:H11, O111:Hnm as well as O157:H7 is notable because the vast majority of previously developed therapeutics against EHEC have focused on O157 only, despite the clinical relevance of the “Big Six” strains. The current incidences across the United States of O26, O111, and O157 are 206, 125, and 807 per 100,000 individuals, respectively (CDC, 2017). Although non-O157 strains are individually less prevalent, the collective contribution of non-O157 strains to GI illness has recently been of growing concern, particularly since surveillance data indicated a 41% increase in the average annual incidence of infection of non-O157 strains over the last 5 years across the United States. The majority of diagnostic, intervention, and awareness strategies have historically been O157-specific (Gill and Gill, 2010; CDC, 2017). O26 and O111 currently account for 25.5 and 15.5% of non-O157 EHEC infections, respectively (CDC, 2017).

In conclusion, we have designed and produced a chimeric antibody with secretory IgA functionality against three EHEC strains and demonstrated its production and assembly in *N. benthamiana*. Testing this antibody to verify that high mannose glycans as opposed to complex glycans usually found on SC interact with the mucus lining of the GI tract will be important toward ensuring *in vivo* functionality. Further work testing the efficacy of V_H_H10-sIgA in live animals will hopefully confirm its ability to prevent EHEC colonization and shedding as well as its utility for fast acting prevention and intervention. Because of its multivalency, V_H_H10-Fc may also be useful if developed as a diagnostic reagent for detecting O26, O111, O145, and O157 in food, the environment, colonized animals, and in infected individuals. Currently, there are no EHEC diagnostics available on the market that can detect both O157 and non-O157 strains despite their clinical relevance. We are optimistic that either of these directions for development will be of value in minimizing EHEC contamination of food and the environment.

## Materials and Methods

### Production of Recombinant EHEC O157:H7 Intimin

A DNA sequence encoding the C-terminal 277 residues of *E. coli* O157:H7 strain ELD933 intimin (Supplementary Figure S1A) was fused C-terminally to MBP and cloned into pMAL-p5X. *E. coli* BL21 (DE3) cells were transformed with the construct and grown overnight under IPTG induction. The next day, cells were harvested, lysed by sonication, centrifuged at 20,000 × *g* for 20 min, and the MBP-Int277 fusion protein was purified using amylose affinity chromatography.

### Isolation of V_H_Hs

Camelid V_H_Hs were generated against recombinant intimin as previously described (Henry et al., 2015, 2016). Experiments involving animals were conducted using protocols approved by the National Research Council Canada Animal Care Committee and in accordance with the guidelines set out in the OMAFRA Animals for Research Act, R.S.O. 1990, c. A.22. Briefly, a male llama (*Lama glama*) was immunized subcutaneously with 180 μg of MBP-Int277 in a total volume of 1 ml Tris-buffered saline (50 mM Tris, 150 mM NaCl, 10% glycerol, pH 8.0) emulsified in an equal volume of complete Freund’s adjuvant (Cedarlane, Burlington, ON, Canada) (day 1). The animal was boosted with the same dose of MBP-Int277 emulsified in incomplete Freund’s adjuvant (Cedarlane) on days 21, 28, 35, and 42. Serum polyclonal antibody responses against MBP-Int277 were monitored using indirect ELISA and detected using HRP-conjugated goat anti-llama IgG antibody (Cedarlane, Cat. No. A160-100P). Total RNA was extracted from peripheral blood lymphocytes collected at days 35 and 49, reverse transcribed, and then expressed V_H_H genes were amplified via two rounds of nested PCR and cloned into the pMED1 phagemid vector. The final size of the phage-displayed V_H_H library size was 5 × 10^6^ independent transformants, with an insertion rate of >95%. Library diversity was verified by DNA sequencing.

V_H_H-displaying phage were rescued from library-containing *E. coli* TG1 cells by coinfection with M13KO7 helper phage and purified by polyethylene glycol precipitation. Library phage were panned for three rounds against 20 μg of MBP-Int277 immobilized in wells of microtiter plates. Bound phage was eluted with 0.1 M triethylamine for 10 min, neutralized with 1 M Tris-HCl, pH 7.4, and amplified in exponentially growing *E. coli* TG1 cells for subsequent panning rounds. After the final round of panning, binding of individual phage clones was assessed by monoclonal phage ELISA and detected using HRP-conjugated rabbit anti-M13 antibody (GE Healthcare, Cat. No. 27-9421-01, Piscataway, NJ, United States).

### Cloning and Transient Expression in *N. benthamiana*

The bovine Fc, JC, and SC sequences were obtained from the NCBI public database (ANN46383, NP_786967, and NP_776568, respectively). The V_H_H_x_-Fc, JC, and SC genes were synthesized by Bio Basic Inc. (Markham, ON, Canada), cloned into pEAQ-DEST-1 plant expression vectors (Sainsbury et al., 2009), and transformed into *A. tumefaciens* (EHA105). *N. benthamiana* plants were grown in a growth chamber at 22°C with a 16 h photoperiod at a light density of 110 μmol m^-2^ s^-1^ for 7 weeks, or in a greenhouse with natural light for 5 weeks before infiltration. Plants were fertilized with water soluble N:P:K (20:8:20) at 0.25 g/l (Plant Products, Brampton, ON, Canada). *Agrobacterium* cultures were prepared as previously described (Saberianfar et al., 2015). Transient expressions were performed either by injection (Miletic et al., 2015) or by vacuum infiltration for small-scale or large-scale transformations, respectively. Prior to vacuum infiltration, *Agrobacterium* transformed with expression vectors encoding either V_H_H3-Fc, V_H_H9-Fc, V_H_H10-Fc, SC, JC, or p19 were sub-cultured from starter cultures and grown separately in Luria-Bertani (LB) broth at 28°C overnight. Each of the cultures bearing constructs encoding the V_H_H_x_-Fc constructs was then combined with cultures carrying SC, JC, and p19. Trays of *N. benthamiana* plants were inverted and submerged into each of these co-cultures and placed into a vacuum chamber. To enable infiltration into the leaves, a pump was used to lower the pressure of the chamber to 85 kPa for 2 min and then immediately released. Plants were transferred back to the growth chamber until sampling.

### Tissue Sampling and Protein Extraction

Depending on the experiment, leaf tissue was collected 4–12 dpi. Four leaf discs were collected from each biological replicate. Protein extraction and total soluble protein quantification were performed as previously described (Conley et al., 2009).

### Recombinant Protein Quantification

Quantification of V_H_H-Fc, JC, and SC was performed by western blot analysis. Samples were run under reducing or non-reducing conditions. Ten micrograms of TSP was resolved using NuPAGE^TM^ 3–8% tris-acetate protein gels (Thermo Fisher Scientific, Waltham, MA, United States) and transferred to PVDF membranes. The recombinant proteins were detected with one of the following primary antibodies: mouse anti-c-Myc monoclonal antibody (GenScript, Cat. No. A00864), mouse anti-HA monoclonal antibody (Millipore Sigma, Cat. No. H3663), mouse anti-FLAG monoclonal antibody (Millipore Sigma, Cat. No. F3165), and HRP-conjugated goat anti-mouse IgG secondary antibody (Bio-Rad, Cat. No. 170-6516). Detection was performed using Enhanced Chemiluminescent detection solution (Biorad Laboratories Inc., Hercules, CA, United States) and a MicroChemi 4.2 imaging system with GelCapture acquisition software (DNA Bio-Imaging Systems Ltd., Jerusalem, Israel). Recombinant proteins were quantified by image densitometry using Totallab TL100 software (Nonlinear Dynamics, Durham, NC, United States), against known amounts of a standard protein loaded on every gel.

### ELISA

ELISAs using plant-produced V_H_H-sIgAs were conducted essentially as described previously (Henry et al., 2015, 2016). Wells of Nunc MaxiSorp^®^ microtiter plates (Thermo-Fisher, Waltham, MA, United States) were coated overnight at 4°C with 100 ng of MBP-Int277 in 35 μl of PBS, pH 7.4. The next day, wells were blocked with 200 μl of PBS containing 2% (w/v) skim milk for 1 h at 37°C. Purified V_H_H-sIgAs were serially diluted in PBS containing 1% (w/v) bovine serum albumin (BSA) and 0.1% (v/v) Tween-20 and added to wells after rinsing 3× with PBS. After incubating for 2 h at room temperature, wells were washed 5× with PBS-T and 2× with PBS. Secondary and/or tertiary antibodies [Monoclonal mouse anti-FLAG^®^ M2 antibody (Sigma–Aldrich, Cat. No. F3165, St. Louis, MO, United States), HRP-conjugated polyclonal donkey anti-mouse IgG (Jackson ImmunoResearch, Cat. No. 715-035-150, West Grove, PA, United States), or HRP-conjugated polyclonal sheep anti-bovine IgA (Abcam, Cat. No. ab12755, Cambridge, United Kingdom)] were diluted 1:5000 in PBS containing 1% BSA and 0.1% Tween-20 and added sequentially to wells, washing 5× with PBS-T and 2× with PBS after each incubation. Wells were developed with 35 μl of tetramethylbenzidine substrate, stopped after 5 min with 35 μl of 1 M H_2_SO_4_, and absorbance at 450 nm was measured using a Multiskan^TM^ FC photometer (Thermo-Fisher).

### Recombinant Protein Purification

Plant extracts were prepared under native conditions as described above. Purification was performed using peptide M/Agarose (Invivogen, San Diego, CA, United States, Cat. No. gel-pdm-5) and anti-DYKDDDDK G1 affinity resin (GenScript, Piscataway, NJ, United States, Cat. No. L00432) according to the manufacturers’ protocols.

### Surface Plasmon Resonance (SPR)

Prior to SPR analyses, V_H_H monomers and MBP-Int277 were purified by size exclusion chromatography using a Superdex^TM^ 75 10/300 GL column (GE Healthcare, Mississauga, Canada) connected to an ÄKTA FPLC protein purification system (GE Healthcare) into HBS-EP+ buffer [10 mM HEPES buffer, pH 7.4, containing 150 mM NaCl, 3 mM EDTA, and 0.05 % (v/v) surfactant P20]. Approximately 700–1600 response units (RUs) of MBP-Int277 were immobilized in 10 mM acetate buffer, pH 4.5, on CM5 sensor chips using an amine coupling kit (GE Healthcare). Multi-cycle kinetic analyses were carried out on a Biacore T200 instrument (GE Healthcare) at 25°C by injecting V_H_Hs at concentrations ranging from 0.3 to 400 nM, at a flow rate of 30–50 μl/min and with a contact time of 300 s, and then allowing the V_H_Hs to dissociate for 600 s. Data were analyzed using BIAevaluation software version 4.1 (GE Healthcare) and fitted to a 1:1 binding model. The MBP-Int277 surface was regenerated between injections using glycine buffer, pH 1.5.

For SPR analyses of plant-produced V_H_H-IgAs, approximately 2200 RUs of V_H_H-IgA or 100 RUs of matched V_H_H monomer were immobilized in 10 mM acetate buffer, pH 3.5, on CM5 Series S sensor chips using an amine coupling kit. Single-cycle kinetic analyses were carried out on a Biacore T200 instrument at 25°C by injecting MBP-Int277 in HBS-EP+ buffer at concentrations ranging from 0.3 to 5 nM, at a flow rate of 30 μl/min and with a contact time of 300–600 s, and then allowing the V_H_Hs to dissociate for 600 s. Data were analyzed using BIAevaluation software version 4.1 (GE Healthcare) and fitted to a 1:1 binding model. The antibody surface was regenerated between injections using glycine buffer, pH 1.5.

### Enterohemorrhagic *E. coli* Binding Assays

Enterohemorrhagic *Escherichia coli* strains O26:H11, O45:H2, O103:H2, O145:Hnm, O121:H19, O111:Hnm, and O157:H7 were obtained from Dr. Michael Mulvey at the Public Health Agency of Canada, National Microbiology Laboratory, *E. coli* Unit, Enteric Diseases Program, Winnipeg, MB, Canada. EHEC strains were individually grown overnight in 5 ml of LB broth (Miller Formulation, Difco, Thermo Fisher Scientific, Ottawa, ON, Canada) at 37°C. The next day, 100 μl of the overnight culture was inoculated in 3 ml of LB broth and grown to an OD_600_ of 0.7–0.9. Cells were harvested from 1 ml of the culture by centrifugation at 13,000 × *g* for 5 min, rinsed three times in PBS for 5 min each time. The bacterial pellet was then resuspended in 1 ml of 2.5% paraformaldehyde (PFA) and incubated at 37°C for 10 min with gentle agitation (350 rpm). The excess PFA was rinsed by centrifugation at 13,000 × *g* for 5 min. The pellet was then resuspended in 200 μl of PBS-T and incubated overnight at 4°C. The next day, 20 μl aliquots of the cell suspension were prepared in separate tubes, centrifuged at 13,000 × *g* for 5 min, and resuspended in 20 μl of the primary plant-produced antibody treatments (100 ng/μl) in PBS-T, as well as PBS-T with no antibody as control, and incubated at 37°C for 90 min with gentle agitation (350 rpm). The primary antibodies were removed by centrifugation at 13,000 × *g* for 5 min, followed by three washes in PBS for 5 min each time. The cells were then resuspended in 20 μl aliquots of secondary antibody, rabbit anti-bovine IgG, IgM, IgA-FITC (1:40 dilution, Thermo Fisher Scientific, Cat. No. SA1-36043), and incubated at 37°C for 1 h. The cells were washed and rinsed three times in PBS as described previously, and one final time in dH_2_O. To stain the bacteria, the cells were resuspended for 2 min in 20 μl aliquots of DAPI (10 mg/ml solution diluted 1:1000 in dH_2_O, Thermo Fisher Scientific, Cat. No. D1306), centrifuged at 13000 × *g* for 5 min, and resuspended in 20 μl of dH_2_O. The cells were then transferred onto poly-L-lysine coated coverslips (Millipore Sigma, Cat. No. S1815) contained in a 24-well plate and centrifuge at 450 × *g* for 10 min. Coverslips were then dried and mounted onto glass slides with Aqua-Poly/Mount (Polyscience Inc., Warrington, PA, United States, Cat. No. 18606).

### HEp-2 Adherence Inhibition Assay

HEp-2 cells were grown in eight-well chamber slides in Dulbecco’s Modified Eagle Medium (DMEM, Life Technologies, Thermo Fisher Scientific, Toronto, ON, Canada) supplemented with 10% fetal bovine serum at 37°C in 5% CO_2_ to ∼80% confluency. EHEC strains O26:H11, O45:H2, O103:H2, O145:Hnm, O121:H19, O111:Hnm, and O157:H7 were individually grown overnight in 5 ml of LB broth at 37°C then subcultured into DMEM at a 1:50 dilution and incubated at 37°C in 5% CO_2_ for 2 h. This subculture was further diluted at 1:10 in DMEM with and without 100 ng/ml of V_H_H10-Fc/SC/JC and then incubated with the HEp-2 cells at 37°C in 5% CO_2_ for 3 h. The cultures were then washed with PBS to remove non-adherent bacteria and fixed using 2.5% PFA (Sigma) in PBS. Cells were then washed in PBS four times and blocked overnight in PBS containing 10% BSA and 0.1% Triton X-100. Cells were then hybridized with Alexa 647 phalloidin (Thermo Fisher Scientific, Cat. No. A22287) used to visualize actin in the HEp-2 cells and donkey anti-rabbit Alexa 350 (Thermo Fisher Scientific, Cat. No. A10039) used to visualize EHEC cells. Cells were then washed in PBS and mounted using Aqua-Poly/Mount (Polyscience Inc., Warrington, PA, United States, Cat. No. 18606). To quantify adherence inhibition by relative fluorescence, the assay was adapted by growing the HEp-2 cells in 96-well black fluorometry plates that had been coated with poly D-lysine. Relative fluorescence was measured using a Synergy2 plate reader (Biotek) using the Gen5 v1.10 software (Biotek). Relative fluorescence of the donkey anti-rabbit IgG Alexa 350 antibody (Thermo Fisher Scientific, Cat. No. A10039) used to visualize EHEC cells was measured in each well at 37°C, with 5 s intermediate shaking, excitation at 360°nm, and emission at 460°nm.

### Confocal and Fluorescence Microscopy

To visualize binding of the V_H_H-sIgA to *E. coli* cells, FITC and DAPI sequential imaging was performed with an Olympus LSM FV 1200 or a Leica TCS SP2 CLSM. Images were acquired with 100× oil objective lens. FITC was imaged by excitation with a 480 nm laser and detection at 520–540 nm. DAPI was imaged by excitation at 350 nm and detection at 455–465 nm. To visualize adherence to HEp-2 cells, a Leica TCS SP2 confocal microscope was used. Images were acquired with a 64× water objective lens. Alexa 647 phalloidin was imaged by excitation at 650 nm and detection at 660–680nm. The donkey anti-rabbit Alexa 350 antibody was visualized by excitation at 350 nm and detection at 455–465 nm.

## Data Availability

All datasets generated for this study are included in the manuscript and/or the Supplementary Files.

## Author Contributions

RM conceived the study. RS and RM designed the research. RS, KH, and AC-F performed the experiments. RS, AC-F, KH, and RM wrote the manuscript. AS assisted with the binding and adhesion experiments. ET provided feedback on experimental design and result interpretations. RS, KH, AC-F, AS, ET, and RM edited the manuscript.

## Conflict of Interest Statement

The authors declare that the research was conducted in the absence of any commercial or financial relationships that could be construed as a potential conflict of interest.

## Supplementary Material

Figure S1 Validation of recombinant maltose-binding protein (MBP)-EHEC O157:H7 intimin fusion protein. **(A)** Amino acid sequence of MBP-Int277 fusion protein. MBP sequence is underlined and TEV protease cleavage site is shown in blue. The fusion protein had a molecular mass of 73,781 Da and a theoretical pI of 6.06. **(B)** SDS-PAGE (4–20% gradient) stained with Coomassie Brilliant Blue. Lane 1, BSA; Lane 2, MBP-Int277. **(C)** Western blot of MBP-Int277 using either anti-6 × His antibody (Lane 3) or anti-MBP antibody (Lane 4). **(D)** Size exclusion profile of MBP-Int277 on a Superdex^TM^ 75 10/300 GL column showing monodisperse behavior. **(E)** Binding of polyclonal goat anti-intimin antibody to MBP-Int277 in ELISA and detected with HRP-conjugated donkey anti-goat IgG.

Figure S2 Sequences for Int277 are similar across EHEC strains O157, O111, O26, and O145. **(A)** Multiple sequence alignment using Clustal Omega default settings of Int277 protein sequence for the seven tested EHEC strains. The alignment has been shaded to show identical residues in black and similar residues in gray. **(B)** Phylogenetic tree using a neighbor joining method to cluster the aligned Int277 sequences based on similarity. The cladogram shown has not been corrected for evolutionary distance and is merely meant to be representative of how the strains cluster based on similarity.
