# Supplementary figures and images for: Plant-Produced Chimeric VHH-sIgA Against Enterohemorrhagic E. coli Intimin Shows Cross-Serotype Inhibition of Bacterial Adhesion to Epithelial Cells
Source: Front Plant Sci. 2019 Mar 12;10:270. doi: 10.3389/fpls.2019.00270 (PMC6445026; doi:10.3389/fpls.2019.00270)

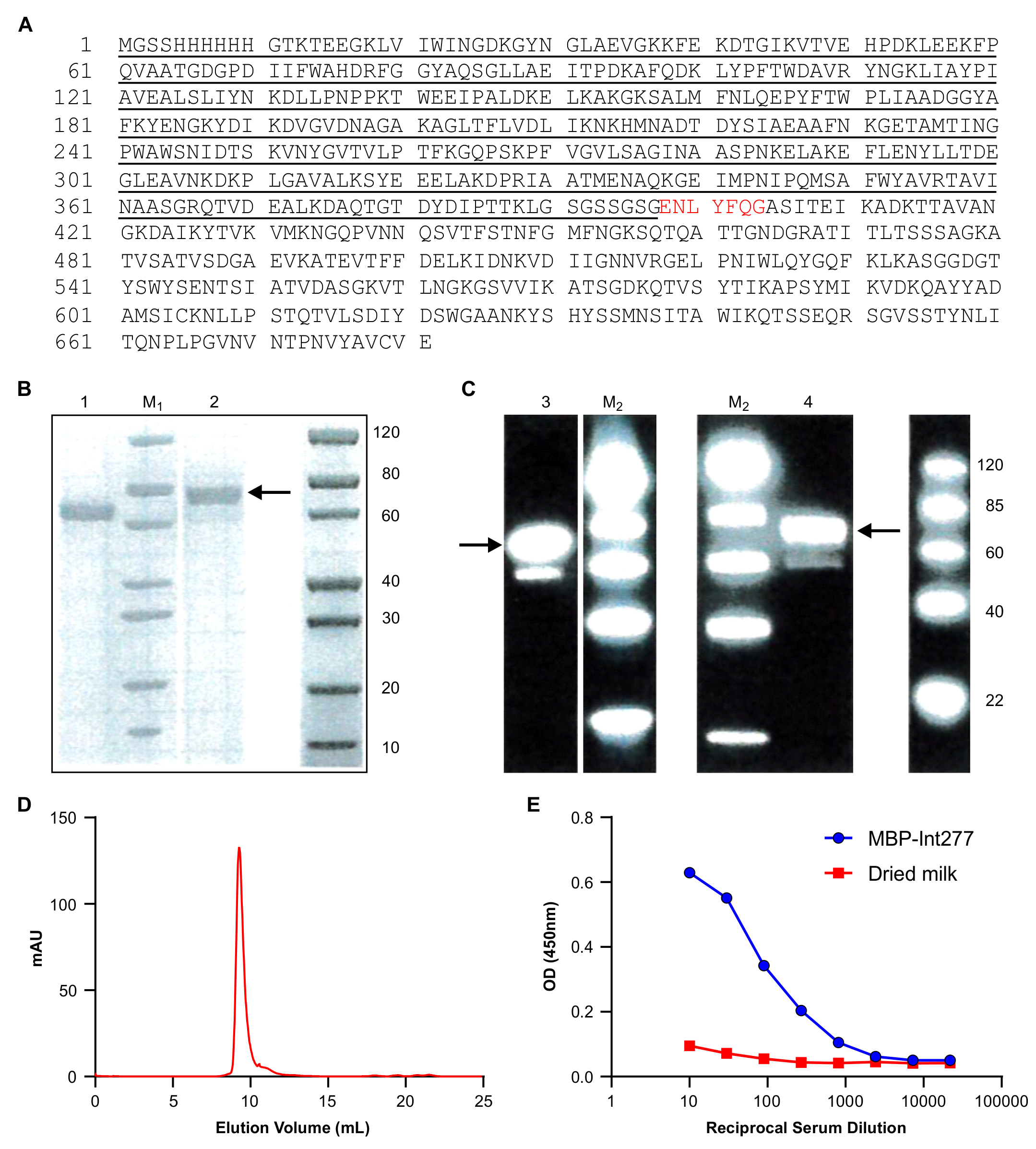

Supplement: Figure S1 — Validation of recombinant maltose-binding protein (MBP)-EHEC O157:H7 intimin fusion protein. (A) Amino acid sequence of MBP-Int277 fusion protein. MBP sequence is underlined and TEV protease cleavage site is shown in blue. The fusion protein had a molecular mass of 73,781 Da and a theoretical pI of 6.06. (B) SDS-PAGE (4–20% gradient) stained with Coomassie Brilliant Blue. Lane 1, BSA; Lane 2, MBP-Int277. (C) Western blot of MBP-Int277 using either anti-6 × His antibody (Lane 3) or anti-MBP antibody (Lane 4). (D) Size exclusion profile of MBP-Int277 on a SuperdexTM 75 10/300 GL column showing monodisperse behavior. (E) Binding of polyclonal goat anti-intimin antibody to MBP-Int277 in ELISA and detected with HRP-conjugated donkey anti-goat IgG. [file Image_1.TIF]

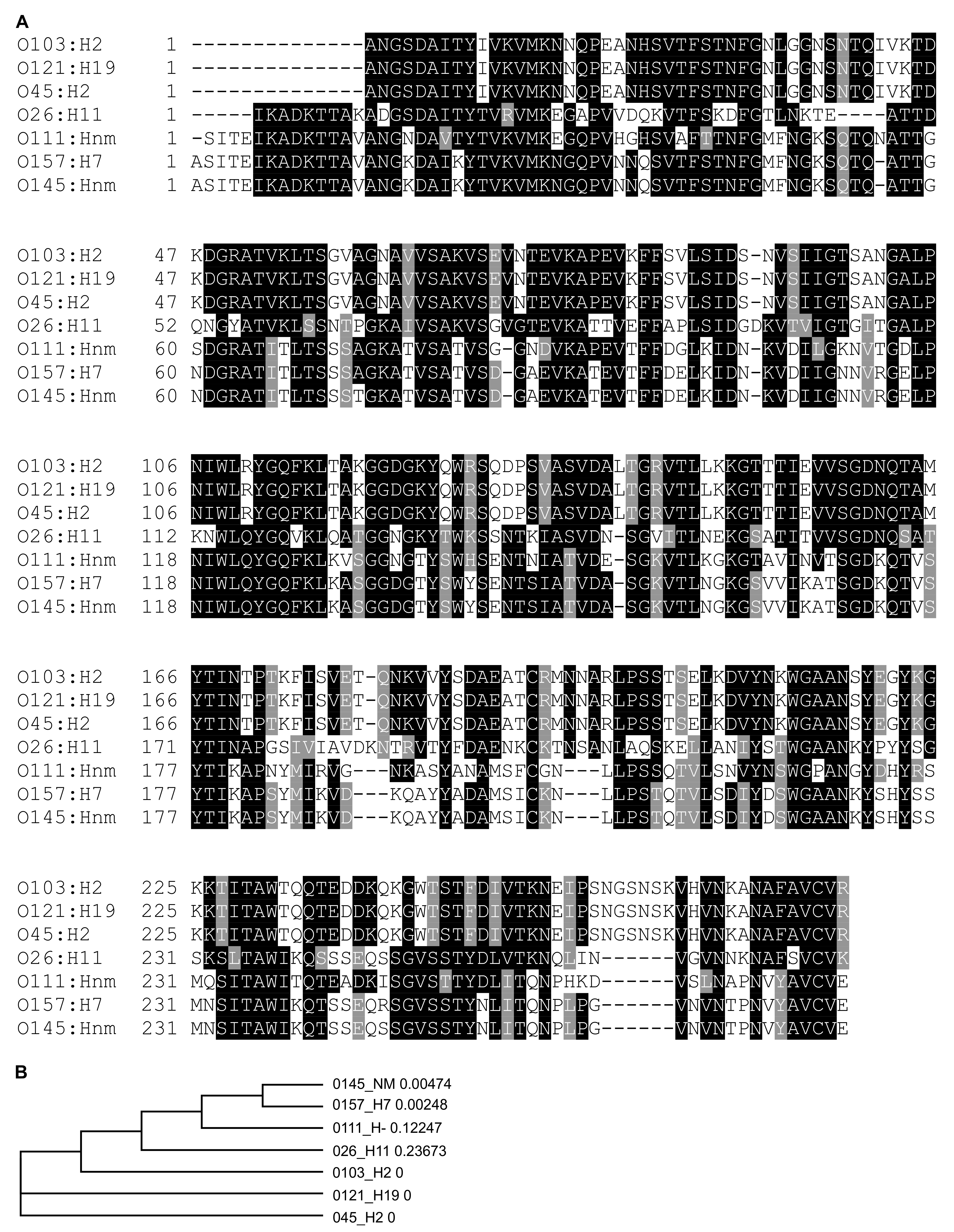

Supplement: Figure S2 — Sequences for Int277 are similar across EHEC strains O157, O111, O26, and O145. (A) Multiple sequence alignment using Clustal Omega default settings of Int277 protein sequence for the seven tested EHEC strains. The alignment has been shaded to show identical residues in black and similar residues in gray. (B) Phylogenetic tree using a neighbor joining method to cluster the aligned Int277 sequences based on similarity. The cladogram shown has not been corrected for evolutionary distance and is merely meant to be representative of how the strains cluster based on similarity. [file Image_2.TIF]
